# Evaluating the association between intravenous immunoglobulin and thromboembolic events in patients with autoimmune diseases: a retrospective real-world study

**DOI:** 10.3389/fphar.2026.1762942

**Published:** 2026-05-26

**Authors:** Jie Chen, Jie Hu, Yanfang Zhang, Suhong Wang, Wenjun Yang, Mingdong Yang, Ziqi Ye, Haibin Dai

**Affiliations:** 1 Department of Pharmacy, The Second Affiliated Hospital, Zhejiang University School of Medicine, Hangzhou, Zhejiang, China; 2 Department of Pharmacy, The Quzhou Affiliated Hospital of Wenzhou Medical University, Quzhou People’s Hospital, Quzhou, Zhejiang, China; 3 Department of Pharmacy, The First Affiliated Hospital, Zhejiang University School of Medicine, Hangzhou, Zhejiang, China; 4 School of Pharmaceutical Sciences, Wenzhou Medical University, Wenzhou, Zhejiang, China; 5 Zhejiang Key Laboratory of Pain Perception and Neuromodulation, Hangzhou, Zhejiang, China

**Keywords:** autoimmune disease (AD), incidence rate, independent risk factors, intravenous immunoglobulin, thromboembolic events

## Abstract

**Background:**

Intravenous immunoglobulin (IVIG), a cornerstone in the management of autoimmune diseases (ADs), entails a concerning risk of thromboembolic events (TEEs). Current risk-stratification models, however, suffer from a critical omission, as they do not include dynamic biomarkers such as D-dimer. Additionally, the effectiveness of intermittent pneumatic compression (IPC) as a physical thromboprophylactic measure before IVIG infusion constitutes an unmet research need. This study was therefore designed to fill these voids by defining the incidence and independent risk factors for IVIG-associated thrombosis in AD patients and by evaluating the prophylactic potential of IPC, with the ultimate goal of informing risk identification and prevention strategies for high-risk patients.

**Methods:**

This study enrolled all AD patients receiving IVIG between January 2022 and October 2024. Patients were grouped by TEE occurrence. We analyzed demographic, clinical, and laboratory data to calculate TEE incidence, identify independent risk factors via logistic regression, and assess the cumulative IVIG dose-TEE association using Kaplan-Meier curves.

**Results:**

The incidence of thromboembolic events was 9.36% (57/609). Multivariate analysis identified these independent risk factors for TEEs: cumulative IVIG dose (OR = 1.00, 95% CI: 1.00–1.00, P = 0.018), age ≥60 (OR = 2.91, 95% CI: 1.52–5.80, P = 0.002), immobility ≥3 days (OR = 6.28, 95%CI: 2.44–20.54, P = 0.001), diagnosis of myelitis (OR = 3.40, 95% CI: 1.01–10.64, P = 0.040) or acute post-transplant rejection (OR = 2.98, 95% CI: 1.02–8.64, P = 0.044), elevated pre-treatment D-dimer (OR = 4.56, 95% CI: 2.14–10.63, P < 0.001), and glucocorticoid use (OR = 2.04, 95% CI: 1.04–4.13, P = 0.041). Pre-treatment IPC showed no protective effect (P = 0.107). A significant dose-response relationship existed, with TEEs increasing markedly above cumulative doses of 100 g.

**Conclusion:**

Independent risk factors for IVIG-associated thrombosis in AD patients included age ≥60, myelitis, transplant rejection, immobility ≥3 days, IVIG dose >100 g, elevated pre-treatment D-dimer, and glucocorticoid use. Prophylactic low-molecular-weight heparin is recommended for these high-risk individuals.

## 1 Introduction

Autoimmune diseases (ADs) are characterized by an immune responses against self-antigens, leading to loss of self-tolerance, tissue injury, and chronic morbidity. These conditions affect an estimated 3%–5% of the global population and impose a substantial disability burden (Wang et al., 2015). Intravenous immunoglobulin (IVIG) serves as a cornerstone therapy for many ADs. Although generally well-tolerated, multiple studies have indicated that IVIG administration significantly increases the risk of thromboembolic events (TEEs), such as myocardial infarction, stroke, and deep vein thrombosis (Go and Call, 2000; Marie et al., 2006; Ammann et al., 2016a). In response to this serious safety concern, the U.S. Food and Drug Administration (FDA) issued a warning regarding TEEs in the prescribing information for IVIG in 2003, which was subsequently upgraded to the agency’s strongest safety alert - a black box warning - in 2013 (Ammann et al., 2016b).

The reported incidence of TEEs associated with IVIG therapy varies considerably across existing literature, ranging from 6.1% to 16.9% (Wittstock et al., 2003; Daniel et al., 2012; Ramírez et al., 2014). Identified risk factors for TEEs also differ among studies. Rajabally et al. suggested that a daily IVIG dose ≥35 g may increase the risk of early thromboembolic complications and recommended caution or consideration of alternative treatments in patients with coronary artery disease, immobility, or four or more risk factors (Rajabally and Kearney, 2011). However, that study included only 62 patients, and this limited sample size may affect the reliability of its conclusions. An analysis by Daniel et al. based on the HealthCore database indicated that advanced age (≥45 years), prior history of TEEs, and hypercoagulable states were associated with an increased thrombotic risk (Daniel et al., 2012). It is noteworthy that findings derived from such administrative databases may have limited generalizability to real-world clinical practice. Furthermore, current risk assessment models exhibit two major limitations: they fail to adequately incorporate key dynamic coagulation biomarkers such as D-dimer, and there is a lack of dedicated studies on the efficacy of intermittent pneumatic compression as physical thromboprophylaxis prior to IVIG treatment. To address these gaps, we conducted a retrospective real-world study. This study aims to systematically evaluate the incidence and independent risk factors of TEEs following IVIG therapy in patients with ADs, with particular emphasis on the analysis of dynamic coagulation biomarkers and the impact of cumulative IVIG dose. Additionally, it seeks to explore effective thromboprophylaxis strategies for high-risk patients, thereby bridging current knowledge gaps in the field.

## 2 Materials and methods

### 2.1 Design of the study

This single-center retrospective study utilized real-world data from eligible patients with ADs who recevied IVIG treatment at the Second Affiliated Hospital of Zhejiang University School of Medicine between January 2022 and October 2024. Data were collected by reviewing electronic medical records (EMRs), including baseline characteristics such as sex, age, body mass index (BMI), primary diagnosis, and relevant medical history. We also extracted medication details-including IVIG dosage, administration rate, and concurrent use of glucocorticoids (e.g., methylprednisolone sodium succinate)-as well as laboratory parameters such as D-Dimer levels. The study was approved by the Institutional Review Board of our hospital (Approval No: 2023-0662). Due to the retrospective and observational nature of the research, the requirement for informed consent was waived. All procedures complied with the principles of the Declaration of Helsinki. Cases were identified using the International Classification of Diseases, 11th Revision (ICD-11).

### 2.2 Patients

Patients diagnosed with ADs and receiving IVIG treatment during hospitalization were identified through the EMR system. Those patients were excluded from the final analysis if they met any of the following criteria: (1) age <18 years; (2) with anticoagulant medications within 14 days prior to IVIG infusion; (3) Presence of arterial or venous thrombotic events (such as cerebral infarction, myocardial infarction, or deep vein thrombosis) upon admission.

### 2.3 Assessment

The primary outcome was defined as the occurrence of a thromboembolic event within the 7-day period starting from the first day of IVIG administration. This definition aligned with the evidence indicating that thrombotic events associated with immunotherapeutic agent-such as IVIG-predominantly occurred within the first week of treatment [2]. All patients were systematically evaluated for TEEs within 7 days following IVIG initiation using one or more of the following imaging modalities: magnetic resonance imaging (MRI) [2], computed tomography angiography (CTA), or color Doppler ultrasound. The primary outcome encompassed TEEs, including deep vein thrombosis, myocardial infarction, and stroke.

### 2.4 Statistical analysis

Continuous variables were presented as medians with interquartile ranges (IQR), while categorical variables were expressed as numbers and percentages. Patients were divided into TEEs group and non-TEEs group based on whether they developed thromboembolic events. For continuous variables, Mann-Whitney U test was used to compare the differences between different groups, while for categorical variables, the Chi-square test or Fisher’s exact test was employed, as appropriate. Besides, multivariate logistic regression models were employed to evaluate potential risk factors. Using the Kaplan–Meier curve with the cumulative IVIG dose as the “time” axis, the cumulative proportion of patients who experienced IVIG-related TEEs under a given cumulative IVIG dose was estimated. For those patients who developed TEEs within 7 days after the first administration of IVIG, the cumulative IVIG doses at the time of thrombosis were recorded. Those patients who did not have TEEs during the study period were censored at the cumulative dose after the last IVIG treatment (Swain et al., 2003). A two-sided P-value <0.05 was considered statistically significant. All statistical analyses were performed using R software 4.3.1 version.

## 3 Rsults

### 3.1 The characteristics of patients

**FIGURE 1:**
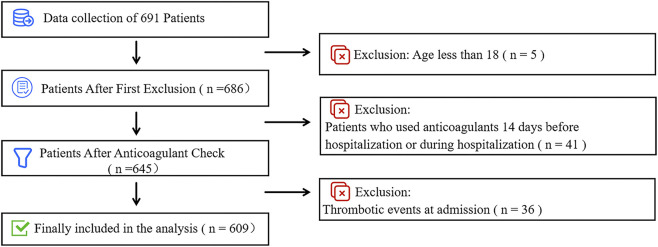
Study screening flow chart.

A total of 691 patients with ADs who received IVIG treatment were initially identified through the EMRs system. Five patients were excluded for being under 18 years of age, 77 were excluded due to receiving anticoagulant therapy within 14 days prior to IVIG treatment or having thrombotic events at admission. Thus, 609 patients were included in the final analysis (Figure 1).

A total of 57 patients experienced TEEs, yielding an incidence rate of 9.4%. The sociodemographic and clinical characteristics of patients were summarized in Table 1. Patients in the TEEs group were significantly older than those in the non-TEEs group (P < 0.001). The proportion of female patients was higher in the TEEs group than in the non-TEEs group (P = 0.045). Additionally, patients in the TEEs group had a significantly higher prevalence of myelitis, chronic inflammatory demyelinating polyneuropathy (CIDP), and acute rejection of solid organ transplantation (AR in SOT) than those in the non-TEEs group (all P < 0.05).

**TABLE 1:** The characteristics of patients.

Characteristics	Non-TEEs group (N = 552)	TEEs group (N = 57)	P-value
Continuous variables, median (IQR)			
Age, years	54 (37–66)	65 (56–70)	**<0.001**
BMI, kg/m^2^	22.8 (20.2–25.2)	23.5 (20.1–25.0)	0.740
Daily dose of IVIG, g	25.0 (20.0–27.5)	25.0 (20.0–30.0)	0.213
Cumulative dose of IVIG, g	112.5 (87.5–137.5)	125.0 (100.0–150.0)	**0.008**
Infusion rate of IVIG, drops/min	40 (30–40)	30 (30–40)	**0.019**
D-dimer, μg/L	350 (220–830)	2050 (853–5,125)	**<0.001**
Categorical variables, n (%)			
Sex, female	276 (50.0)	20 (35.1)	**0.045**
IPC	76 (13.8)	3 (5.3)	0.107
Concomitant use of methylprednisolone	243 (44.0)	40 (70.2)	**<0.001**
Comorbidity			
Diabetes	98 (17.8)	6 (10.5)	0.232
Malignant tumor	35 (6.3)	6 (10.5)	0.260
HF	24 (4.3)	5 (8.8)	0.178
CVA	28 (5.1)	2 (3.5)	>0.999
Hepatic insufficiency	56 (10.1)	6 (10.5)	>0.999
Renal insufficiency	20 (3.6)	3 (5.3)	0.467
COPD	13 (2.4)	3 (5.3)	0.182
Previous VTE	18 (3.3)	2 (3.5)	0.710
Hyperlipidemia	47 (8.5)	2 (3.5)	0.302
Hypertension	168 (30.4)	18 (31.6)	0.978
Bedridden status ≥3 days	273 (49.5)	51 (89.5)	**<0.001**
Indication			
Myelitis	19 (3.4)	7 (12.3)	**0.007**
Neuromyelitis optica	18 (3.3)	2 (3.5)	0.710
AIDP	184 (33.3)	12 (21.1)	0.082
Myasthenia gravis	94 (17.0)	5 (8.8)	0.156
AE	105 (19.0)	14 (24.6)	0.407
AR in SOT	14 (2.5)	12 (21.1)	**<0.001**
PAN	5 (0.9)	0 (0.0)	>0.999
Pulmonary infection (immunocompromised patients)	32 (5.8)	5 (8.8)	0.378
DM/PM	7 (1.3)	0 (0.0)	>0.999
CIDP	41 (7.4)	0 (0.0)	**0.025**
CTD	10 (1.8)	0 (0.0)	0.610
ITP	21 (3.8)	0 (0.0)	0.246
Sepsis	2 (0.4)	0 (0.0)	>0.999

AE, Autoimmune encephalitis; AIDP: Acute inflammatory demyelinating polyneuritis; AR in SOT, Acute rejection of Solid Organ Transplantation; CIDP, chronic inflammatory demyelinating polyneuritis; CTD, Connective-tissue disorder; CVA, Cerebrovascular accident; HF: Heart Failure; DM/PM: Dermatomyositis/ Polymyositis; ITP, immune thrombocytopenic purpura; IPC, Intermittent pneumatic compression; PAN, Polyarteritis Nodosa. VTE, Venous Thromboembolism. Bold values indicate statistically significant differences (P < 0.05) between the TEEs and non-TEEs groups.

### 3.2 Independent risk factors for thrombotic events by multivariate logistic regression

We further established a multivariate logistic regression model to examine the relationship of TEEs with nine potential risk factors, including older age, female sex, higher cumulative IVIG dose, faster IVIG infusion rate, prolonged immobility (defined as bedridden status ≥3 days) during treatment, diagnoses of myelitis, or acute solid organ transplant rejection, concurrent glucocorticoid use, and elevated D-dimer levels within 7 days prior to treatment. The variable for CIDP was excluded from the multivariate analysis owing to its complete absence in the TEEs group, which precluded a meaningful assessment of its association with TEEs.

Multivariate analysis demonstrated that the cumulative dose of IVIG was an independent risk factor for TEEs (OR = 1.00, 95% CI: 1.00–1.00, P = 0.018), along with age ≥60 years (OR = 2.91, 95% CI: 1.51–5.80, P = 0.002), bedridden status ≥3 days (OR = 6.28, 95% CI: 2.44–20.54, P = 0.001), diagnosis of myelitis (OR = 3.40, 95% CI: 1.01–10.64, P = 0.040), acute rejection of solid organ transplantation (OR = 2.98, 95% CI: 1.02–8.64, P = 0.044), elevated D-dimer levels within 7 days before treatment (OR = 4.56, 95% CI: 2.14–10.63, P < 0.001), and concomitant glucocorticoid use (OR = 2.04, 95% CI: 1.04–4.13, P = 0.041) (Table 2).

**TABLE 2:** Independent risk factors for TEEs by multivariate logistic regression.

Variables	OR	95% CI	P-value
Cumulative dose of IVIG, g	1.00	1.00–1.00	0.018
Infusion rate of IVIG, drops/min	0.99	0.95–1.03	0.701
Age, years			
<60	Ref	​	​
≥60	2.91	1.51–5.80	0.002
Sex (female vs. male)	0.62	0.31–1.20	0.160
Bedridden status ≥3 days (yes vs. no)	6.28	2.44–20.54	0.001
Myelitis (yes vs. no)	3.40	1.00–10.64	0.040
AR in SOT (yes vs. no)	2.98	1.02–8.64	0.044
Concomitant use of methyl-prednisolone (yes vs. no)	2.04	1.04–4.13	0.041
D-dimer			
<500	Ref	​	​
≥500	4.56	2.14–10.63	<0.001

AR in SOT, Acute rejection of Solid Organ Transplantation; CI, Confidence Interval; OR, Odds Ratio.

### 3.3 Thrombotic events and cumulative incidence by cumulative IVIG dose

**FIGURE 2:**
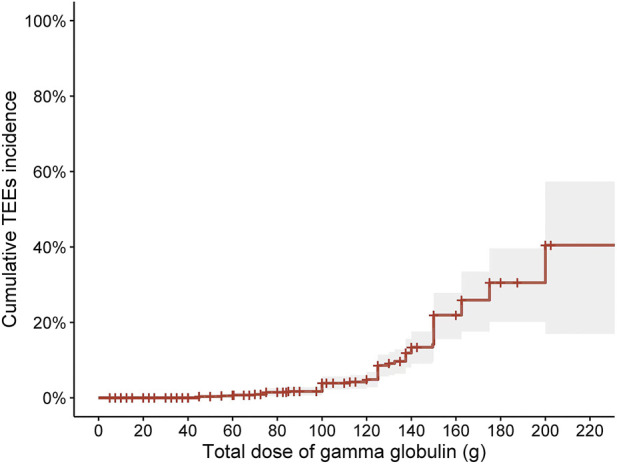
Cumulative TEEs Incidence by Gamma Globulin Dose: Kaplan-Meier Analysis. The cumulative dose of IVIG at the time of thrombotic events among 609 randomly selected patients who received IVIG treatment.

As detailed in Table 3, the number of patients at risk, total TEEs, and the estimated cumulative incidence of TEEs are presented for specified cumulative IVIG dose levels. The majority of TEEs occurred at cumulative IVIG doses ≥100 g, with a cumulative incidence of 3.92% (95% CI: 2.17–5.64). As the cumulative dose increased to 125 g, the incidence rose significantly to 8.59% (95% CI: 5.64–11.45), and further increased to 21.91% (95% CI: 15.52–27.82) at 150 g. These trends were corroborated by Kaplan-Meier analysis (Figure 2), which demonstrated a progressive elevation in the cumulative incidence of TEEs with increasing total IVIG dose. Notably, pronounced increases in incidence were observed beyond the thresholds of 100 g, 125 g, and 150 g. Together, these findings support a positive dose-response relationship between cumulative IVIG exposure and the risk of TEEs.

**TABLE 3:** Estimated cumulative percentage of patients with TEEs, by cumulative IVIG dose.

Cumulative IVIG dose, g	The number of patients at risk	The number of TEEs cases	Cumulative incidence rate (%)	95% CI (%)
45.0	555	2	0.36	0.00–0.86
55.0	546	1	0.54	0.00–1.15
60.0	541	1	0.73	0.01–1.43
70.0	511	1	0.92	0.11–1.72
75.0	504	3	1.51	0.47–2.55
85.0	469	1	1.72	0.60–2.83
100.0	447	10	3.92	2.17–5.64
112.5	329	1	4.21	2.37–6.01
120.0	293	2	4.86	2.82–6.87
125.0	281	11	8.59	5.64–11.45
130.0	167	1	9.14	6.01–12.16
132.5	165	1	9.69	6.39–12.87
137.5	163	4	11.90	8.00–15.64
140.0	117	2	13.41	9.01–17.59
149.5	112	1	14.18	9.55–18.58
150.0	111	10	21.91	15.52–27.82
162.5	39	2	25.92	17.53–33.46
175.0	32	2	30.55	20.14–39.60
200.0	7	1	40.47	16.94–57.34

CI, Confidence Interval.

In the study population, the incidence of TEEs was associated with the cumulative IVIG dose (Figure 2). The cumulative probability of developing IVIG-associated TEEs was 1.72% at a cumulative dose of 85 g, 3.92% at 100 g, and 4.21% at 112.5 g. The cumulative incidence demonstrated a gradual, dose-dependent increase rather than a sharp threshold effect. However, the slope of the curve increased notably around the cumulative dose level of 100 g.

## 4 Discussion

The immune and coagulation systems exhibit close interconnection. In immune-mediated diseases, multiple mechanisms can significantly increase the risk of thrombosis, such as a shift in the inflammation-driven hemostatic balance toward a procoagulant state (Zöller et al., 2012). As a core therapeutic approach for ADs, IVIG has been documented in multiple studies to be associated with thrombotic adverse events in clinical practice (Orbach et al., 2005; Hamrock, 2006; Guo et al., 2018). However, considerable discrepancies exist in the reported incidence of IVIG-associated TEEs across different studies. For instance, a 2014 study by Ramírez et al. reported a 16.9% incidence of thrombotic events, including stroke, myocardial infarction, and pulmonary embolism, among 303 IVIG-treated patients [8]. In contrast, a large-scale retrospective study by Daniel et al. based on the HealthCore database (including 11,785 patients) documented a TEEs incidence of only 1% (Daniel et al., 2012). In the present study, which included 609 patients, we observed a TEEs incidence of 9.4%. This intermediate value may reflect potential ethnic or regional variations in the clinical manifestations of IVIG-associated thrombosis. Notably, all thrombotic events in our cohort were DVTs, whereas European and American studies more frequently report arterial events such as stroke and myocardial infarction-a distinction that further supports the above inference.

Previous studies on risk factors for IVIG-associated TEEs have primarly focused on treatment-ralated parameters (e.g., dosage, infusion rate) and patients’ underlying comorbidities (e.g., cardiovascular history, advanced age) (Sridhar et al., 2014), while the predictive value of coagulation and fibrinolysis biomarkers has remained relatively underinvestigated. Through systematic analysis, our study identified that elevated D-dimer levels within 7 days prior to IVIG administration were significantly and positively associated with the risk of TEEs. As a specific degradation product of cross-linked fibrin, D-dimer directly reflects the degree of fibrinolytic system activation, and its fluctuation is closely related to coagulation cascade, activation, fibrinolysis, and inflammatory status (Weitz et al., 2017; Borowiec et al., 2020). Notably, the research team led by Simes previously confirmed that persistently elevated D-dimer levels are significantly associated with increased TEEs risk (Simes et al., 2018), a finding that aligns with our results and further supports the use of D-dimer as an effective biomarker for assessing hypercoagulable states and predicting thrombosis. In addition to novel biomarkers, our study also validated the importance of conventional risk factors. Advanced age and prolonged immobilization, well-established risk factors for Venous thromboembolism (VTE) (Wilkerson and Sane, 2002; Cao et al., 2021), were similarly confirmed as independent predictors of IVIG-associated TEEs in our cohort. From a pathophysiological perspective, progressively worsening age-related blood hypercoagulability and continuous decline in vascular endothelial function collectively form the fundamental basis for the increased incidence of TEEs in elderly patients (Yamamoto et al., 2005). This mechanism has also been corroborated by multiple clinical observations—compared with younger populations, elderly patients indeed demonstrate a significantly higher incidence of TEEs following IVIG treatment (Elkayam et al., 2000; Byrne et al., 2002; Caress et al., 2003; Alexandrescu et al., 2005; Chun et al., 2021). Beyond thrombotic risk, older adults are generally more vulnerable to adverse drug events and potentially inappropriate medications, which underscores the importance of individualized risk assessment in this population (Zou et al., 2025). To address the potential confounding effect of disease-related immobility, we included “bedridden status ≥3 days” as a covariate in the multivariate analysis. As shown in Table 2, prolonged immobility was a strong independent risk factor for TEEs, while cumulative IVIG dose remained independently significant after this adjustment. This supports a specific contribution of IVIG to thromboembolic risk, independent of immobility.

The thrombogenic mechanism of IVIG is primarily attributed to its induction of a state of blood hyperviscosity. A study on high-dose IVIG provides supporting evidence: all patients exhibited increased serum viscosity following infusion, which peaked at approximately 1.37 times the upper limit of normal and subsequently declined to baseline levels within 1 month (Dalakas, 1994). Multiple reports have indicated that the occurrence of TEEs is associated with the loading dose of IVIG, and exceeding the recommended dosage elevates thrombotic risk (Brown and Ballas, 2003; Katz and Shoenfeld, 2005). This study demonstrates that even when IVIG is administered according to recommended regimens, the cumulative dose remaines an independent risk factor for TEEs, prompting further investigation into potential supratherapeutic thresholds. Kaplan-Meier analysis revealed that the incidence of IVIG-related TEEs increased with higher cumulative doses: the risk remaines relatively low and stable at cumulative doses below 100 g, whereas surpassing successive thresholds of 100 g, 125 g, and 150 g is associated with a significant stepwise increase in incidence. Additionally, no significant difference was observed between the two groups in the use of pneumatic compression devices for thromboprophylaxis before treatment (P = 0.107). Based on these findings, patients requiring high-dose IVIG therapy—particularly those with a cumulative dose ≥100 g—should undergo an individualized thrombotic risk assessment before initiating treatment. For patients identified as high-risk, prophylactic anticoagulation with low-molecular-weight heparin prior to IVIG infusion may be beneficial.

This study has several limitations. First, the relatively low incidence of TEEs may have limited the statistical power, potentially hampering our ability to identify all relevant risk factors; a larger sample size would allow for a more precise estimation of their independent effects. Second, due to the retrospective nature of the study, there was substantial missing data for key coagulation parameters—such as thrombin time and prothrombin time—which precluded their inclusion in the multivariate analysis. Third, certain inflammatory conditions such as vasculitis or sepsis may inherently increase thrombotic risk. Although we adjusted for disease indication in the multivariate model, residual confounding cannot be completely excluded, and future prospective studies with larger sample sizes are needed to disentangle disease-related from IVIG-related thrombogenicity.

## 5 Conclusion

In this retrospective real-world study of patients with ADs receiving IVIG therapy, several factors were identified as potential independent risk factors for thromboembolic events, including age ≥60 years, diagnosis of myelitis or acute rejection following solid organ transplantation, prolonged immobility (bedridden status ≥3 days), cumulative IVIG dose exceeding 100 g, elevated pre-treatment D-dimer levels, and concurrent glucocorticoid use. Prophylactic anticoagulation with low-molecular-weight heparin may be considered for such high-risk patients. Given the retrospective nature and potential for residual confounding, these findings should be interpreted with caution, and further prospective validation is warranted.

## Data Availability

The original contributions presented in the study are included in the article/supplementary material, further inquiries can be directed to the corresponding authors.
